# Changes in Host Response to *Mycobacterium tuberculosis* Infection Associated With Type 2 Diabetes: Beyond Hyperglycemia

**DOI:** 10.3389/fcimb.2019.00342

**Published:** 2019-10-04

**Authors:** Cristian Alfredo Segura-Cerda, Wendy López-Romero, Mario Alberto Flores-Valdez

**Affiliations:** ^1^Doctorado en Farmacología, Universidad de Guadalajara, Guadalajara, Mexico; ^2^Biotecnología Médica y Farmacéutica, Centro de Investigación y Asistencia en Tecnología y Diseño del Estado de Jalisco, Guadalajara, Mexico

**Keywords:** *Mycobacterium tuberculosis* infection, type 2 diabetes, hyperglycemia, diabetic dyslipidemia, hormones

## Abstract

Tuberculosis (TB) remains as the first cause of death among infectious diseases worldwide. Global incidence of tuberculosis is in part coincident with incidence of type 2 diabetes (T2D). Incidence of T2D is recognized as a high-risk factor that may contribute to tuberculosis dissemination. However, mechanisms which favor infection under T2D are just starting to emerge. Here, we first discuss the evidences that are available to support a metabolic connection between TB and T2D. Then, we analyze the evidences of metabolic changes which occur during T2D gathered thus far for its influence on susceptibility to *M. tuberculosis* infection and TB progression, such as hyperglycemia, increase of 1AC levels, increase of triglycerides levels, reduction of HDL-cholesterol levels, increased concentration of lipoproteins, and modification of the activity of some hormones related to the control of metabolic homeostasis. Finally, we recognize possible advantages of metabolic management of immunity to develop new strategies for treatment, diagnosis, and prevention of tuberculosis.

## Introduction

Infection with *Mycobacterium tuberculosis*, which in susceptible people leads to either active or latent tuberculosis (TB), remains as a high-burden health problem globally. It is calculated that in 2017, TB caused 1.3 millions deaths, and 10.0 million new cases were reported (WHO, 2018). Some disorders have been recognized as risk factors to develop pulmonary TB, such as HIV coinfection, malnutrition, tobacco smoking, and type 2 diabetes (T2D) (WHO, 2018).

T2D is a chronic metabolic disorder that essentially affect the function of pancreatic β-cells, resulting in progressive development of insuline resistance and chronic inflammation (Defronzo et al., 2015). Several meta-analysis show that T2D is associated with a two- to four-fold increased risk of active TB, even multidrug-resistant TB (Amare et al., 2013; Al-Rifai et al., 2017; Liu et al., 2017; Hayashi and Chandramohan, 2018). As a risk factor for developing TB, T2D has attracted attention by its projected increase and its prevalente worldwide. In 2018, it was estimated that T2D affected one of each 10 individuals globally (425 million people around the world) and it is projected that the number of cases of T2D may increase by 40% in 2045 (IDF, 2017). Hence, an increase in the global burden of T2D poses a higher risk of TB spread worldwide in the upcoming years.

Epidemiological data about comorbidity between TB and T2D show that there is a close relationship between both diseases. A recent meta-analysis showed that 16% of newly-diagnosed TB patients have T2D and up to 4.1% of T2D patients develop TB (Wilkinson et al., 2017). In 2017, close to 800,000 newly-diagnosed TB cases were attributed to T2D, and T2D was the risk factor that contributed to most TB cases in countries like China and India, even above HIV infection (WHO, 2018). However, the incidence of TB-T2D can be higher than reported, because the American Association of Diabetes (ADA) estimates that around 50% of T2D patients remain undiagnosed and the WHO has found higher rates of undiagnosed TB mainly in low-income countries (American Diabetes Association, 2018a; WHO, 2018). Both WHO and the International Union against Tuberculosis and Lung Disease recommend to diagnose T2D in newly-diagnosed TB patients, with the aim of generating pharmacological strategies that effectively contribute to the clinical management of T2D and TB (Liu et al., 2019).

**Figure 1:**
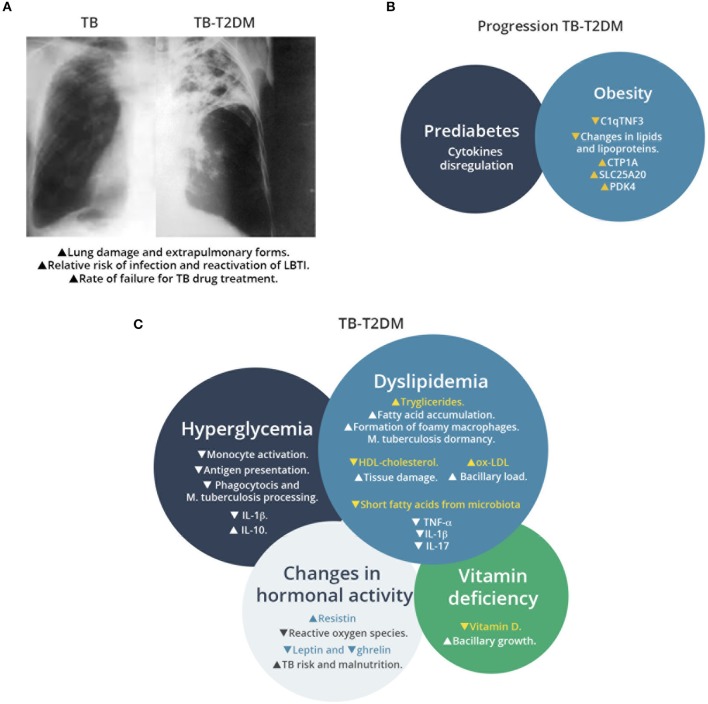
Overview of TB-T2D comorbidity, factors associated to T2D that increase susceptibility to *M. tuberculosis* infection, and metabolic changes that affect the host immune response. **(A)** Schematic representation of chest radiography of *M. tuberculosis* infected lung in patients with or without T2D. Comorbidity increases the lung damage associated to *M. tuberculosis* infection. **(B)** Factors associated to progression of TB in T2D patients. Prediabetes and obesity contribute to TB susceptibility with progressive changes in T2D patients, as recent signatures for TB progression have revealed recently. **(C)** Effects of metabolic changes in TB-T2D on the host response *to M. tuberculosis* infection. TB-T2D patients suffer hyperglycemia, dyslipidemia, changes in hormonal activity and vitamin deficiency. These conditions result in higher manifestations of *M. tuberculosis* infection.

In addition to its importance on TB prevalence, patients with T2D have more severe manifestations of TB than non-T2D ones (Carreira et al., 2012; Figure 1A) and have a lower response to anti-TB treatment (OR 2.93) (Viswanathan and Gawde, 2014) compared to patients without T2D. Moreover, the recurrence and reactivation of LTBI, which potentially contributes to TB dissemination, is higher in T2D patients than in non-T2D ones (OR = 1.83) (Jimenez-Corona et al., 2013).

Some relevant innate and adaptive immunity mechanisms that are necessaries for *M. tuberculosis* clearance have been shown to be compromised in patients with TD2M, as recently reviewed (Restrepo, 2016; Kumar Nathella and Babu, 2017; Rao et al., 2019). However, a causal relationship between T2D and TB has not been completely addressed. Even though the role of hyperglycemia on the immunity against *M. tuberculosis* has been widely described (Magee et al., 2018), new evidences about other metabolic changes that occur during T2D progression are emerging, such as the effects of dyslipidemia, vitamin deficiency, and changes in hormonal activity, which will be discussed in specific sections below. Here, we discuss diverse findings ranging from *in vitro* studies to clinical research, which support the connection between T2D progression and TB susceptibility in a metabolic-dependent way and present a global connection of feasible events that may occur during this comorbidity, which might create an appropriate environment to *M. tuberculosis* infection, and we also present evidences of the role of the treatment of T2D on this response.

## Prediabetes and Progression to TB

Prediabetes is defined as a stage where glucose levels do not meet with the criteria for T2D but are too high to be considered as normal. Prediabetes is diagnosed by a fasting blood glucose (FBG) values between 100 and 125 mg/dL or 2 h oral glucose tolerance test (OGTT) between 140 and 199 mg/dL or Hemoglobin 1AC between 5.7 and 6.4% (American Diabetes Association, 2019a). Even though a time-regulated interaction between T2D and TB remains unresolved, some evidences suggest that progression from prediabetes to T2D may influence the susceptibility to *M. tuberculosis* infection (Figure 1B). An study of household contacts of active TB patients (who had a higher risk to develop TB) showed that prediabetes is present in at least 27% of them (Shivakumar et al., 2018), while another study in western India that included 1,073 participants, revealed that more than one-half of newly diagnosed TB patients had T2D or pre-T2D (Mave et al., 2017).

Prediabetes induces changes in cytokines production that are related to control of *M. tuberculosis* infection. A study in prediabetic-TB patients showed that they had higher circulating concentrations of IFN-γ, TNF-α, IL-12, IL-17, IL-1β, GM-CSF (cytokines that favors Th1 response) and also had higher concentrations of IL-5, IL-10, and TGF-β (cytokines related to regulation of cytokine response) than TB patients without prediabetes (Kumar et al., 2014). This dysregulation of cytokines levels found in plasma may compromise the immune response against *M. tuberculosis*, and suggest that progressive changes in immune response related to T2D progression may influence the susceptibility to TB.

Progression of T2D in obese patients produces changes that can be related to an increased TB susceptibility through modulation of adypocytokines such as the C1q tumor necrosis factor related protein-3 (C1qTNF3 or CTRP-3). C1qTNF3 is a cytokine produced by macrophages and adipocytes that reduces inflammation generated by adipocytes (Kopp et al., 2010; Schmid et al., 2014). A study in obese patients showed that T2D induces a reduction in the plasmatic concentrations of this cytokine as compared with non-T2D patients (Elsaid et al., 2019). A study conducted in South Africa and Gambia, showed that patients who progress from a Latent TB Infection (LTBI) to active TB (LTBI defined as TST+, Quantiferon TB assay+) have lower concentrations of C1qTNF3 in plasma than LTBI patients who did not progress to TB during a more than 1 year follow-up (Penn-Nicholson et al., 2019). These findings suggest that the reduction in C1qTNF3 might be a potential contributor to the increased risk for TB in people with T2D, and might be a factor worth evaluating in the clinic.

In formally established T2D (as opposed to pre-T2D), some additional disorders in metabolism may occur, such as hyperglycemia, dyslipidemia, changes in lipoprotein concentrations, and changes in hormonal profiles (Olokoba et al., 2012; Carrera Boada and Martinez-Moreno, 2013). These alterations seem to be an adequate environment for *M. tuberculosis* infection to thrive in T2D subjects, likely improving persistence of mycobacteria and allowing them to consolidate the pulmonar infection and its effects (Figure 1C).

## Clinical Features of T2D and Their Effects on Response to *M. tuberculosis* Infection

### Effect of Hyperglycemia on Susceptibility to *M. tuberculosis* During T2D

T2D is characterized by an increase in blood concentration of glucose (measured as FBG >126 mg/dL or measured as 2 h postprandial glucose >200 mg/dL), known as hyperglycemia, and higher proportions of 1AC in blood (>6.5%) (American Diabetes Association, 2018a).

High glucose concentration in blood has been related to defects in host response against *M. tuberculosis* infection. An study in Japan, where FBG levels in 522 TB-confirmed patients were tested, showed a positive association (+1.31) between glucose intolerance and the development of TB (OR 3.15, C.I. 1.12–8.88; Hayashi et al., 2014). This positive association was also found in an a cross-sectional study in African individuals, where subjects with random blood glucose (RBG) concentration ≥11.1 mmol/L had 2.15 times the odds of prevalent TB than individuals with a RBG concentration <11.1 mmol/L (Bailey et al., 2016).

Rationale dissection of the effect of hyperglycemia indicates that it can affect the activity of macrophages. Macrophages are one of the first cells to encounter mycobacteria and are the main cells where mycobacteria resides during infection (Srivastava et al., 2014).

Montoya-Rosales and collaborators (Montoya-Rosales et al., 2016) cultured monocyte-derived macrophages from human U-937 cell line in the presence of different concentrations of glucose in the medium, and observed a negative correlation between glucose concentration and phagocytosis of *M. tuberculosis*. In the same system, the authors described a positive correlation between glucose concentration and induction of LL-37 antimicrobial peptide, and with the induction of anti-inflammatory cytokines such as TGF-β and IL-10 (Montoya-Rosales et al., 2016).

Another defect in macrophage function caused by hyperglycemia is the expression of receptors related to antigen presentation and related to T cell activation. A study where alveolar macrophages from T2D patients were infected with *M. tuberculosis* H37Rv, reported reduced levels of expression of CD86, CD80, and HLA-DR, molecules that participate in antigen presentation and Th response; and also a reduced induction of IL-6, IL-1β, IL-10, and IL-12 before and after the infection (Lopez-Lopez et al., 2018). Taken together, the works by Montoya-Rosales et al. (2016) and Lopez-Lopez et al. (2018) suggest a potential mechanistic explanation that contribute to partially explain the influence of hyperglycemia on the susceptibility to M. tuberculosis infection in T2D patients, but studies *in vivo* are needed to clearly establish the role of hyperglycemia on TB during TB-T2D comorbidity.

Glycemic control is commonly measured by hemoglobin 1AC because it represents the concentration of glucose found in blood 2 or 3 months before the actual measurement has been done (American Diabetes Association, 2018a). A glycemic level over the recommended by ADA (1AC <7%, preprandial capillary glucose between 83 and 130 mg/dL and peak postprandial capillary plasma glucose <180 mg/dL; American Diabetes Association, 2019b) has been correlated with higher prevalence of TB among T2D patients (Almeida-Junior et al., 2016) and with extended lung damage caused by TB, detected by computed tomography (Xia et al., 2018), or by radiological examinations (Huang et al., 2017) in patients with TB-T2D. However, a mechanistic explanation about the role of 1AC in promoting lung pathology in TB-T2D remains undescribed.

Regarding the role of an increase in 1AC levels and its effects on innate immune response, a study in India determined the levels of monocyte activator markers (CD164, sCD163, and CRP) in plasma of TB, T2D, T2D, and healthy controls (Kumar et al., 2019). In this study, 1AC of either TB or TB-T2D patients was positively correlated with levels of these markers. In addition, authors demonstrated that in patients who were diabetic before developing TB, the levels of these markers were increased compared with those of healthy controls, suggesting that changes in monocyte activation marker profiles coincident with changes in 1AC may be progressive. In another study conducted in Indonesia, cytokines in blood were determined in two defined groups of patients depending on their 1AC levels (<7 and >7%). Patients with 1AC >7% had the same levels of IFN-γ and IL-12 than the patients with 1AC <7%, but had lower levels of vitamin A (Ginandjar et al., 2016). Deficiency in vitamin A levels has been recently associated to a higher risk of TB in Moroccan patients (Qrafli et al., 2017), and this vitamin was found in lower levels in TB patients than in healthy controls (Oh et al., 2017). Whether or not there is a correlation between low 1AC levels and decreased vitamin A (or vice versa) remains to be determined.

Even though 1AC levels have not been found to be related to anti-TB treatment success in both T2D and non-T2D patients (Tabarsi et al., 2014), recent evidence suggest that higher levels of 1AC in TB patients may produce a delayed time to sputum conversion in TB patients receiving anti-TB treatment (Nurwidya et al., 2018). More efforts are needed to better understand the relationship between 1AC and the susceptibility to *M. tuberculosis* infection.

### Effect of Diabetic Dyslipidemia on Susceptibility to *M. tuberculosis* During T2D

T2D is associated with some alterations in lipid of lipids and lipoproteins, a condition described as diabetic dyslipidemia (Carmena, 2008). This disorder comprises high levels of triglycerides, reduction of high-density lipoprotein (HDL) cholesterol concentrations and high levels of low-density lipoproteins (LDL) in blood (Wu and Parhofer, 2014; Cuevas et al., 2016).

A recent analysis by ^1^H-Nuclear Magnetic Resonance of lipid metabolic markers (lipoproteins, fatty acids, glycerides, amino acids, and glycolytic molecules) in plasma samples from TB, T2D, and TB-T2D patients showed that TB-T2D comorbidity displays characteristics of both diseases, such as reduced concentration of amino acids (indicator of waste) and lower dyslipidemia than expected only in T2D (Vrieling et al., 2018). In the same study, authors recognized that there were differences in the profiles among individuals, perhaps attributable to the chronicity of T2D and the time lapse of comorbidity TB-T2D. This suggested that there is a dynamism between both diseases that generates differences in lipid environments, which, in turn, could lead to different responses against infection.

As for the effects of triglycerides on TB, an *in vitro* study using PBMC-derived macrophages and THP-1 derived macrophages, showed that *M. tuberculosis* incorporates triglycerides from the host to infection sites in conditions of hypoxia (Daniel et al., 2011; Guerrini et al., 2018). This condition creates a fatty acid-rich environment for *M. tuberculosis* that favor the use of lipids as the major source of energy during *M. tuberculosis* infection, as documented in granulomas in rabbits, marmosets, and humans (Guerrini et al., 2018). A recent systematic review and meta-analysis has reinforced this concept by finding a positive correlation between latent infection by *M. tuberculosis* (defined as TST+ or IGRA+) with T2D, OR = 1.18 (1.06–1.30; Lee et al., 2017).

Accumulation of lipids in macrophages leads to formation of cytokine secreting foam cells in T2D patients. A study where J774 murine macrophages were exposed to serum of T2D patients, showed that serum triglycerides levels correlated with induction of lipid accumulation in these cells and consequently, in the formation of foamy macrophages (Cui et al., 2010). It is known that formation of foamy macrophages contributes to persistence of bacteria and to the tissue pathology during TB (Russell et al., 2009). Nevertheless, the direct effect of high levels of triglycerides in T2D patients on the formation of foamy macrophages during TB infection has not been addressed in clinical studies.

The observed reduction in HDL-cholesterol in T2D patients, has also been observed in patients with pulmonary tuberculosis (Deniz et al., 2007; Rao, 2009). This reduction in HDL-cholesterol was recently identified as a risk factor for severe lung lesions in patients with TB-T2D (Dong et al., 2018). However, the role of the reduction in HDL-cholesterol on the host response against *M. tuberculosis* is still unaddressed, and need to be explored in future studies.

Further to the above mentioned increase in concentration of triglycerides in T2D patients, they had higher concentrations of oxidized-LDL (Ox-LDL) in serum than healthy controls (Vrieling et al., 2019). In guinea pigs, it has been demonstrated that ox-LDL accumulates in lungs during active infection by *M. tuberculosis* (Parish et al., 2012), which is in line with findings *in vitro* that indicates that ox-LDL is taken up by macrophages in environments rich in glucose (Hayek et al., 2005). The effect of ox-LDL on bacilli activity was addressed by Parish and collaborators, who performed a study using alveolar macrophages obtained from guinea pigs loaded with Ox-LDL and then infected with *M. tuberculosis H37Rv*. They observed that Ox-LDL-treated macrophages had higher bacillary loads than untreated macrophages after 3 days of culture (Parish et al., 2012). Coincidentally, this effect has also been observed in primary human macrophages exposed to Ox-LDL before infection with *M*. *tuberculosis* H37Rv, where there was an increase of the bacterial burden in a dose-dependent manner within macrophages *in vitro* (Vrieling et al., 2019). These evidences reinforce the fact that the higher concentrations of Ox-LDL found in T2D patients may contribute to a higher susceptibility to TB progression and suggest that diabetic dyslipidemia is closely related to TB susceptibility.

## Physiopathological Mechanisms Altered in T2D and Their relationship With TB Susceptibility

### Hormones Related to Blood Glucose Control and Their Possible Roles During TB-T2D

Leptin and ghrelin are two hormones related to control of blood glucose concentration that are related to malnutrition during TB (Chang et al., 2013; Mexitalia et al., 2017). In a study where the concentration of leptin and ghrelin in blood was measured in TB, TB-T2D, and healthy Chinese people, there were found lower levels of leptin in TB-T2D patients as compared with TB patients, and higher levels of ghrelin were observed in TB-T2D patients than in TB patients (Zheng et al., 2013). These data suggest that T2D may increase susceptibility to TB through affecting appetite through these changes in ghrelin and leptin, which are related to a higher susceptibility to *M. tuberculosis* infection (Buyukoglan et al., 2007).

Resistin is a protein suggested to be part of the development of insulin resistance in humans and mice (Benomar et al., 2013), and it has been recognized as a key molecule that links obesity and T2D (Steppan et al., 2001). Resistin participates in increasing expression of proinflammatory cytokines such as TNF-α, IL-6, IL-12, and monocyte chemoattractant protein (MCP)-1 in PBMCs, macrophages, and hepatic stellate cells via the nuclear factor-κB (NF-κB) pathway (Bokarewa et al., 2005). It was shown that patients with T2D had higher levels of resistin in serum and this increase correlated with a diminished capability of THP-1 human macrophages to induce the production of reactive species of oxygen (ROS) *in vitro* against a challenge with *M. tuberculosis* (Chao et al., 2015). Another study in patients without T2D, found higher concentrations of serum resistin in TB patients than in healthy controls, and this concentration diminishes during 6 months of treatment with a standard anti-tuberculosis treatment (Ehtesham et al., 2011). These evidences suggest TB induces a metabolic change in the production of resistin, which has effects in both metabolic and immunologic response and derives in macrophage defective functions.

Production of intestinal incretins, proteins released after food intake and which stimulate insulin secretion (Nauck and Meier, 2018), such as GLP (glucagon-like peptide) is reduced in patients with T2D (Mannucci et al., 2000). GLP is degraded by the enzyme dipeptidyl peptidase IV (DPP-4) and as a part of the antidiabetic treatment, blockers of DPP4 are commonly used for glucose control (Deacon, 2019). A recent study found a negative correlation in blood levels of DPP4 and the chemoattraction of cells to lung in TB infected humans (Blauenfeldt et al., 2018). According to this study, TB patients had a lower amount of DPP4 than healthy controls and this was correlated with TB pathology through the recruitment of Th1 T cells to the site of infection. Even though the effect on blood glucose levels through the use of DPP4 blockers during TB-T2D on the host response to *M. tuberculosis* infection has not been addressed, the hormonal control of appetite and blood glucose may cause modulation of the of immune response.

### Modifications in Microbiota in T2D Patients and Its Role on Immunity Against Mycobacteria

Patients with T2D have modifications in the composition of their intestinal microbiota that may lead to tuberculosis susceptibility (Zhang et al., 2013). In T2D patients, there is a significant reduction in the numbers of bacteria that produces short-chain fatty acids (SCFA) (Larsen et al., 2010; Morrison and Preston, 2016). The role of SCFA in response to *M. tuberculosis* was explored *in vitro* by Lachmandas and collaborators by exposing isolated Peripheral Blood Mononuclear Cells (PBMCs) to SCFA (acetate, propionate or butyrate) and measuring cytokines that participate in the proinflammatory and anti-inflammatory response against a stimulus with *M. tuberculosis* H37Rv lysates *in vitro*. Treatment with SCFA decreased induction of TNF-α, IL-1β, and IL-17, while it did not modify the induction of IL-6, IFN-γ, or IL-22 (Lachmandas et al., 2016). A reduced production of TNF-α or IL-1β is related with higher bacillary burden, while a reduced production in IL-17 has been related to lower migration of T cells to the site of infection, conducting to severe scenarios of infection (Domingo-Gonzalez et al., 2016). Even though Lachmandas and collaborators did not demonstrate that SCFA concentrations affected bacillary load in this *in vitro* model, it was clear that they affected host response against *M. tuberculosis* infection and left open the opportunity to experimentally address their influence *in vivo* using suitable models.

### Vitamin D Deficiency in T2D and Its Effect on Immune Response Against Mycobacteria

Vitamin D is known to play a role in the control of TB infection (Kearns and Tangpricha, 2014). In addition, vitamin D is related with the control of blood glucose in T2D though modulation of insulin resistance and insulin secretion (Norman et al., 1980). Vitamin D deficiency is common in T2D patients, and some studies have related this deficiency with an increased risk of TB. A recent study has correlated the presence of pre-T2D and T2D with lower levels of vitamin D in TB patients in China (Zhao et al., 2017). Another study in recently diagnosed TB patients in China, either with TB or TB-T2D, revealed that there is an association between diminished vitamin D levels in serum and TB (OR = 3.26, C.I. 1.56–6.82) or TB-T2D (OR = 2.27, C.I. 1.05–4.92) (Wang et al., 2017).

Regarding the evidences linking vitamin D deficiency to a higher risk of TB, an study in Tanzania, revealed that vitamin D deficiency is related to an increased risk of TB only in patients with persistent hyperglycemia (OR = 4.0, C.I. 0.86–18.54) (Boillat-Blanco et al., 2016), and another study in China showed that patients with more than 10 years of T2D diagnosis have lower levels of vitamin D (Zhao et al., 2018). In addition to these data, an study in Chinese patients showed that both T2D and pre-T2D people have lower levels of vitamin D in serum (Zhao et al., 2017). These results suggest that there is a time-dependent relationship between vitamin D, hyperglycemia and TB risk.

A study in Mexico, where monocytes obtained from T2D patients (with confirmed vitamin D deficiency) were infected with *M. tuberculosis* H37Ra, showed that bacterial replication was higher in monocytes from T2D patients than in monocytes from healthy controls (Herrera et al., 2017), suggesting that vitamin D (among other possible defects as already described above) contribute to reduced control of *M. tuberculosis* in cells derived from T2D patients.

## Treatment of T2D and its Impact on TB Susceptibility

### Effect of Anti-hyperglycemic Drugs on the Host Response to *M. tuberculosis* Infection

According to ADA, metformin is the preferred initial treatment for hyperglycemia in T2D patients (American Diabetes Association, 2018b) and, even though a recent systematic review found evidence that suggest the glycemic control may have a favorable effect on anti-TB treatments outcomes (Shewade et al., 2017), its influence on the host response against *M. tuberculosis* is controversial.

Two recent retrospective studies in Taiwan showed that the treatment of hyperglycemia with metformin is a factor that prevents TB in T2D patients [HR 0.552, 95% C.I. 0.493–0.617 (Tseng, 2018) and HR 0.84, 95% C.I. 0.74–0.96 (Lee M. C. et al., 2018)]. In spite of its protective role, there is no conclusive information about the effectiveness of adjunctive therapy with metformin and anti-TB treatment. A recent report suggest that metformin acts by reducing concentration of circulating metalloproteinases (MPP-1, -2, and -8), and it is correlated with reduced bacterial burden in TB-T2D patients treated with metformin and antibiotics (Kumar et al., 2018). However, a study conducted in Seoul showed that treatment of TB-T2D patients with metformin plus the anti-TB treatment, did not have an effect on the sputum culture conversion nor TB recurrence within 1 year after treatment completion (Lee Y. J. et al., 2018), which is in line of findings of not increased efficacy of TB treatment by administration of metformin in mice (Dutta et al., 2017). These evidences suggest that control of hyperglycemia may contribute to the maintenance of a metabolic environment that may be able to reduce the susceptibility to TB throughout mechanisms that act independently of antibiotic activity.

A study where PBMCs from healthy controls were cultured with glibenclamide, a sulphonylurea also used to reduce hyperglycemia, produced a reduction in M1 markers (CD14+ CD16–), and increased M2 markers (CD14+ CD16+) in these cells independently of infection with BCG. Moreover, when PBMC obtained from subjects who were treated with oral glibenclamide were infected with *M. tuberculosis*, they exhibited an impaired capability to kill bacilli (Kewcharoenwong et al., 2018). While to date, there are no studies reported that correlate the use of glibenclamide with TB susceptibility or anti-TB treatment efficacy, some studies in healthy subjects have demonstrated that there are pharmacological interactions between glibenclamide and glimepiride (another sulphonylurea used to control of hyperglycemia) (Surekha et al., 1997; Niemi et al., 2000). Future studies in this regard are needed to clarify the role of glibenclamide on TB treatment efficacy.

### Effect of Pharmacological Treatment of, or Vaccination With BCG, on Diabetic Dyslipidemia and Host Response to *M. tuberculosis* Infection

As we described above, some metabolic changes associated with diabetic dyslipidemia can be actively implicated in the susceptibility of the host to *M. tuberculosis* infection. Treatment of dyslipidemia has been shown to influence the capability to respond to *M. tuberculosis* infection. Lee and collaborators identified in a retrospective study that Taiwanese individuals with T2D and treated with statins had a lower risk of developing active TB (RR 0.76, 95% C.I. 0.60–0.97), than individuals with T2D and no treatment with statins (Lee et al., 2015). However, treatment of dyslipidemia had no influence on the efficacy of a combined therapy formed by antibiotic-treatment and statins or fibrates against TB, as a cohort study showed recently (Chen et al., 2019). These evidences suggest that control of diabetic dyslipidemia should have different effects on the host response to *M. tuberculosis* depending on the stage of TB, before activation of infection, or when infection is established.

Vaccination with BCG produces changes on parameters related to diabetic dyslipidemia. An study where guinea pigs were vaccinated or not with BCG, showed that vaccination produces a lower accumulation of Ox-LDL at the site of infection after challenge with *M. tuberculosis* H37Rv than accumulation observed in unvaccinated guinea pigs (Parish et al., 2012). This effect regarding the accumulation of ox-LDL is in agreement with results obtained with peritoneal macrophages isolated from hyperlipidemic APOE*Leiden. CETP mice vaccinated with BCG, which after vaccination, had lower concentrations of non-HDL cholesterol than unvaccinated mice, and this event correlated with lower formation of foamy cells (Van Dam et al., 2016). These data suggest that immunization with BCG has also an effect on lipid metabolism and may contribute to the protection afforded against TB by pathways other than those directly involved in immune responses.

## Concluding Remarks

Recently, a systematic review showed that more than 70% of new drug efficacy trials have not considered the T2D population in their protocols (Lutfiana et al., 2019). We strongly believe research in TB should encompass efforts to understand how the presence of risk factors like T2D may compromise the efficacy and/or immunogenicity of vaccines, or affect the sensibility of diagnostic methods, or even the therapeutic effect of new drugs.

As a mathematical modeling of TB-T2D shows, control of T2D may lead to a significant reduction in TB incidence, and the uncontrolled raise of T2D should have the opposite effect (Pan et al., 2015). We think that a better understanding of the mechanisms that couple metabolic changes with TB susceptibility will lead to the design of better therapies, which would focus on the management of the TB-T2D comorbidity. We do not rule out the use of drugs that are traditionally used in the T2D treatment as a host-directed therapy against TB, as metformin and statins, even though further studies are needed to fully ascertain their efficacy as a complement for the treatment of TB. Furthermore, we suggest that the design and evaluation of current and future vaccine candidates against TB, or diagnostic methods that aim to early diagnose TB before the onset of overt disease in T2D patients, should consider including their evaluation in T2D preclinical models, as well as in human volunteers whenever a clinical trial is designed and performed.

T2D patients undergo some metabolic modifications that may facilitate the establishment, maintenance, and progression of *M. tuberculosis* infection. Since the onset of a prediabetic stage, the metabolic environment compromises the host response and produces changes that allows infection and favors disease progression and its worsening. In this sense, identification of biomarkers of progression to TB in prediabetic patients is needed to better understand the transition point between progression or not to TB. Recent efforts to elucidate a metabolic signature of progression to TB revealed that some metabolic networks may be related to progression to TB (Duffy et al., 2019). In particular, genes that encode for molecules that participate in fatty-acid metabolism and protein catabolism resulted as significantly associated to TB progression: *CTP1A*, which codes for carnitine palmitoyltransferase 1A was downregulated in obesity (Orellana-Gavalda et al., 2011); *SLC25A20*, which codes for carnitine acyl-carnitine translocase and has been observed to contribute to insulin secretion in mouse models of obesity (Soni et al., 2014); and *PDK4*, which codes for pyruvate dehydrogenase kinase, a key enzyme that participates in control of blood glucose in an insuline-dependent manner (Lee, 2014), and is increased in diabetic rats (Wu et al., 1998).

During T2D and TB comorbidity, the basal levels of glucose, 1AC, triglycerides, HDL-cholesterol, lipoproteins, and hormones related to the control of metabolic parameters creates an environment that supports bacterial survival and their spread (Figure 1). Finally, we contend that a better understanding of how immunity is modulated by metabolic signatures, will allow us to identify targets for treatment of TB-T2D patients, which lead to an improved control of T2D and resolution of TB disease by an enhanced clearance of *M. tuberculosis* from infected cells.

## Author Contributions

CS-C and MF-V extensively discussed the manuscript. All authors wrote the manuscript, reviewed drafts, and approved submission of this work.

### Conflict of Interest

The authors declare that the research was conducted in the absence of any commercial or financial relationships that could be construed as a potential conflict of interest.
